# Use of Octopus™ Tissue Stabilizer for Minimal Manipulation Approach of Bronchial Anastomosis in Lung Transplant

**DOI:** 10.21470/1678-9741-2022-0413

**Published:** 2023-09-11

**Authors:** Mauro Razuk Filho, Samuel Lucas dos Santos, Flavio Pola dos Reis, Luis Gustavo Abdalla, Lucas Matos Fernades, Paulo Manuel Pêgo-Fernandes

**Affiliations:** 1 Lung Transplant Group, Disciplina de Cirurgia Torácica, Hospital das Clínicas, Faculdade de Medicina, Universidade de São Paulo, São Paulo, São Paulo, Brazil

**Keywords:** Coronary Artery Bypass, Cardiopulmonary Bypass, Heart-Lung Machine, Hemodynamics, Lung Transplantation, Treatment Outcome

## Abstract

Bronchial anastomotic complications are a cause of grave concern for surgeons that perform lung transplantations. There are several risk factors that may lead to this complication, being inadequate surgical technique one of them, specifically regarding adequate exposure and manipulation of the bronchial stump and anastomosis. Here we report the use of Octopus™ Tissue Stabilizer as a mean to allow for a better exposure of the stump and facilitate a “no-touch” approach towards anastomosis. Systematic application of devices that facilitate the employment of the correct surgical techniques can have an effect in reducing the incidence of bronchial anastomotic complications.

## INTRODUCTION

**Table:** 

Abbreviations, Acronyms & Symbols	
BAC	= Bronchial anastomotic complications
VA-ECMO	= Venoarterial extracorporeal membrane oxygenation

Since lung transplantation became a validated treatment for advanced lung diseases, bronchial anastomotic complications (BAC) have been a cause of serious concern for surgeons. Over the past decades, several advances have been made in identifying and controlling the risk factors that may lead to such complications.

The medical literature reports an incidence of 1.3%-33.0% of BAC following lung transplantation. Factors such as ischemia, infection, and lung ischemic time are well documented elements that predispose to BAC. Nevertheless, the surgical technique adopted is equally important for the successful healing of the anastomosis. One aspect that surgeons should take into consideration is the minimal manipulation of the bronchial stump in order to preserve the peribronchial tissue intact. This “no-touch” technique associated with the use of end-to-end anastomosis and wrapping the bronchus with a vascularized tissue (omentum, pericardium, muscle flap) assuredly reduce the risk of BAC^[1-3]^.

The Octopus™ Tissue Stabilizer (Medtronic, Inc, Minneapolis, Minnesota, United States of America) is a device conceived for cardiac surgery that uses a flexible arm to stabilize the surgical site for an off-pump coronary artery bypass grafting. The implementation of cardiac devices is not new in the world of lung transplantation. Several authors demonstrated how heart apical suction devices can provide a better exposure of the left hilum^[4,5]^. However, few have utilized such devices aiming at the reduction of BAC. With that in mind, we added the Octopus™ Tissue Stabilizer to the repertoire of weapons employed in order to decrease the manipulation of the stump and allow for better postoperative results.

## TECHNIQUE

Following the usual preoperative procedures, we initiated the transplantation with exposure of both lungs and the mediastinum by bilateral anterolateral thoracotomies and transverse sternotomy in the fourth intercostal space (“clamshell” incision). There were no pleuro-pulmonary adherences, nor were there pleural effusion or lymph nodes. Central venoarterial extracorporeal membrane oxygenation (VA-ECMO) was installed due to previous pulmonary hypertension.

**Fig. 1:**
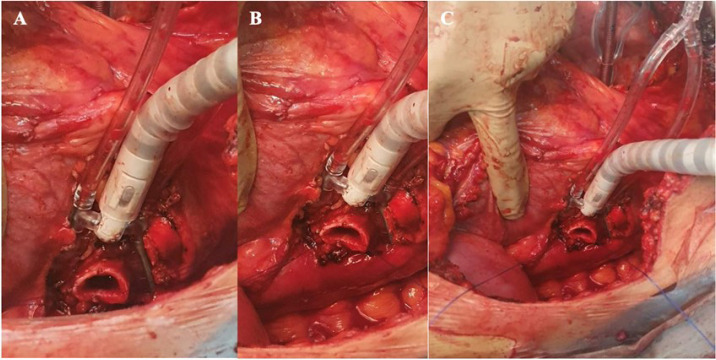
Exposure of the left bronchial stump using the Octopus™ Tissue Stabilizer (Medtronic, Inc, Minneapolis, Minnesota, United States of America). A) Left bronchial stump exposed; B) two anchor sutures are attached to the extremities of the bronchial stump, allowing its traction; C) view of the left thoracic cavity after exposure and traction of the left bronchial stump.

The patient was then subjected to sequential bilateral lung transplantation, starting with the left lung. After pneumectomy, the left bronchial stump was exposed using Medtronic Octopus™ Tissue Stabilizer, attached to Finochetto retractor, to allow minimum manual manipulation of the stump (Figure 1). There was no need to use the suction mechanism. After carefully dissecting the stump, we performed an end-to-end anastomosis using 3-0 monofilament nonabsorbable polypropylene running suture. The anastomosis was then wrapped in a pericardial flap. Subsequently, we began the arterial and venous anastomoses, with 5-0 and 4-0 monofilament nonabsorbable polypropylene suture, respectively. After deairing of the suture lines, both anastomoses were completed, and controlled ventilation and reperfusion of the left donor lung was started. The same process was repeated for the right lung.

The gas flow of the VA-ECMO was then gradually reduced, and the arterial blood gas and cardiac function were evaluated, permitting decannulation. Four chest drains were placed: two 36Fr posteriorly and two 28Fr anteriorly bilaterally, and the chest wall was closed in the usual manner. The patient remained in VA-ECMO for a total of four hours and 30 minutes.

The ischemic times were 280 minutes on the left side and 380 minutes on the right side. The patient was extubated on the first postoperative day and subjected to a bronchoscopy on the 15^th^ postoperative day and showed no signs of BAC. The patient tolerated a gradual reduction of supplementary oxygen and was discharged breathing without the need of auxiliary oxygen on the 23^rd^ postoperative day.

## DISCUSSION

As surgeons, we are always aiming to improve our technique as a mean of reducing complications and providing a better care to our patients. In complex situations such as lung transplants, the adequate exposure of the hilar structures is imperative for performing bronchial anastomosis. Adopting a “no-touch” or “minimal touching” technique while handling the bronchial stump reduces BAC. Nonetheless, for that to occur, one requires a superb exposure of the bronchial stump.

The Octopus™ Tissue Stabilizer is a validated device for cardiac surgery as a tissue stabilizer for off-pump coronary artery bypass grafting. It utilizes vacuum at the extremity of its “U” shaped claw to soothe the heartbeat on a designated spot and assist the cardiac surgeon. Our idea was to employ the distal claw of such device to stabilize the bronchial stump and perform the bronchial anastomosis with a “no-touch” technique. As we did not have a need to soothe the heartbeat, the suction mechanism was kept turned off.

It is important to report that in this case, as in others, several other techniques were applied to reduce BAC - use of end-to-end anastomosis and pericardial flap. Therefore, it is impossible to affirm that the application of the Octopus™ Tissue Stabilizer is responsible for the favorable outcome of this patient.

## CONCLUSION

In short, we believe that the systematic application of devices that facilitate the employment of the correct surgical techniques can have an effect on reducing the incidence of BAC as well as allowing for less invasive approaches, while providing no additional risk to the patient.

## Abbreviations, Acronyms & Symbols

BAC: Bronchial anastomotic complications
VA-ECMO: Venoarterial extracorporeal membrane oxygenation
